# A study on volatile organic compounds emitted by in-vitro lung cancer cultured cells using gas sensor array and SPME-GCMS

**DOI:** 10.1186/s12885-018-4235-7

**Published:** 2018-04-02

**Authors:** Reena Thriumani, Ammar Zakaria, Yumi Zuhanis Has-Yun Hashim, Amanina Iymia Jeffree, Khaled Mohamed Helmy, Latifah Munirah Kamarudin, Mohammad Iqbal Omar, Ali Yeon Md Shakaff, Abdul Hamid Adom, Krishna C. Persaud

**Affiliations:** 1 Centre of Excellence for Advanced Sensor Technology, Universiti Malaysia Perlis, Arau, Perlis Malaysia; 2 Cell and Tissue Engineering Lab (CTEL), Department of Biotechnology Engineering, Kulliyyah of Engineering, International Islamic University Malaysia (IIUM), Kuala Lumpur, Malaysia; 3 International Institute for Halal Research and Training (INHART), International Islamic University Malaysia (IIUM), Kuala Lumpur, Malaysia; 4 Department of Respiratory, Hospital Tuanku Fauziah, Jalan Kolam, Kangar, Perlis Malaysia; 5 School of Chemical Engineering and Analytical Science, University of Manchester, Oxford Road, Manchester, United Kingdom

**Keywords:** E-nose, In-vitro, GCMS-SPME, Lung cancer, VOCs

## Abstract

**Background:**

Volatile organic compounds (VOCs) emitted from exhaled breath from human bodies have been proven to be a useful source of information for early lung cancer diagnosis. To date, there are still arguable information on the production and origin of significant VOCs of cancer cells. Thus, this study aims to conduct in-vitro experiments involving related cell lines to verify the capability of VOCs in providing information of the cells.

**Method:**

The performances of e-nose technology with different statistical methods to determine the best classifier were conducted and discussed. The gas sensor study has been complemented using solid phase micro-extraction-gas chromatography mass spectrometry. For this purpose, the lung cancer cells (A549 and Calu-3) and control cell lines, breast cancer cell (MCF7) and non-cancerous lung cell (WI38VA13) were cultured in growth medium.

**Results:**

This study successfully provided a list of possible volatile organic compounds that can be specific biomarkers for lung cancer, even at the 24th hour of cell growth. Also, the Linear Discriminant Analysis-based One versus All-Support Vector Machine classifier, is able to produce high performance in distinguishing lung cancer from breast cancer cells and normal lung cells.

**Conclusion:**

The findings in this work conclude that the specific VOC released from the cancer cells can act as the odour signature and potentially to be used as non-invasive screening of lung cancer using gas array sensor devices.

## Background

Cancer is one of the leading causes of mortality among humans worldwide. These phenomena are mainly because cancer commonly detected at a very late stage. The American Cancer Society [1], estimated about 1,685,210 new cases of cancer to be diagnosed and 595,690 cancer related deaths to be reported in the United States in the year 2016. It is also reported that lung cancer (LC) is the second most common cancer affecting men (14%) and women (13%) behind only prostate cancer (21%) and breast cancer (29%) respectively [1]. In Malaysia, LC has been reported to be the second most common cancer affecting men and the third most common cancer affecting females with 2,100 Malaysians diagnosed each year [2].The diagnosis of lung cancer at an early stage, particularly when the tumour is discovered at its local site, has been shown to improve the survival rate of patients [3, 4]. Hence it is critical that high risk patients are screened. However, the established and widely used screening techniques, such as chest radiography and cytological examination, often give poor results in detecting small and resectable cancers [5].

Currently, the application of low dose computed tomography (LDCT) as an early stage lung cancer screening technique shows reduction in the number of lung cancer-based deaths [6]. Yet, this method exposed patients to great risk as the high amount of radiation used can lead to several complications [4, 7]. Generally, conventional methods are invasive and might delay the therapy if the cancer is found [8, 9]. In addition, only selected hospital with the right expertise and facilities can perform such screening tests. Thus, a new screening approach based on the cell biology theory [4] using the analysis of volatile organic compounds (VOCs) linked to lung cancer has been receiving considerable attention from researchers. This new screening technique is non-invasive, reliable and inexpensive [10, 11].

The change in metabolic pathways (gene or protein changes) in cancerous cells during tumour growth may lead to peroxidation of the cell membrane and production of certain VOCs [12, 13]. These VOCs can be detected directly on the headspace of the cancer cells [8, 14], or exhaled breath of cancer patients [10, 15, 16]. In the case of exhaled breath air, VOCs generated by the cancer cells are released by blood and exchanged through the alveolus in the lung [17]. The potential of detection of VOCs in the breath of lung cancer patients to be used as diagnostic or screening tools have been extensively analysed and studied for several years [18]. However, in order to provide cellular and biochemical origin information of VOCs to clinicians for the decision on the specific treatment for the cancer, the analysis should also be compared with cancer cells (0either in-vivo or in-vitro) [19, 20].

Many studies of in-vitro cultured cells as a model system to demonstrate the discrimination between tumour and normal cells using spectrometric technique have been reported [21–32]. However, the results are somewhat equivocal and more studies are essential to identify VOC biomarkers of lung cancer [32]. There are only few studies conducted using an array of sensors to distinguish types of lung cancer cells based on in-vitro cultured cell lines samples [8, 33, 34] as shown in Table 1. These reports show substantial results in term of performance of the sensors. However, the use of the right classification algorithms for e-nose performance with the aid of SPME-GCMS analysis is crucial to strengthen the findings and progress the aim of non-invasively cancer diagnosis [35, 36].

**Table 1:** List of sensor array used, cell lines, cell growth time considered for measurement (incubated period) and type of matrix used in in-vitro studies aiming to distinguish type of lung cancer by previous studies

Sensor Type	Cell lines	Control	Incubation (hours)	Matrix Type	Statistical Approach	Reference
GNP	NSCLC: A549, Calu-3, H1650, H4006, H1435, H820 and H1975	Pure medium	-	Culture medium with cells	PCA	[8]
GNP	NSCLC: A549, Calu-3, H1650, H4006, H1435, H820, H1975, H2009, HCC95, HCC15, H226 and NE18 SCLC: H774, H69, H187, and H526	IBE, pure medium	68	Culture medium with cells	Linear nu-SVC-SVM; cross validation	[33]
C320	NSCLC: L55, L65, A549, H460, M51 and REN	NHDF and HASM	-	Cells in saline solution	MD	[34]

In this study, the VOCs signature of the two types of lung cancer cell lines which are A549 and Calu3 will be investigated. The normal lung cell line and the breast cancer cell line are used as control samples to differentiate the lung cancer-related VOCs. As to date, no known reported work investigating VOC patterns released by both lung and breast cancer cultured cell lines under the same conditions, environment and at different growth stages.

This paper presents new results distinguishing the VOCs generated by two types of cancer cell lines, namely lung cancer (A549 and Calu-3) and breast cancer (MCF7), as well as normal lung (WI38VA13) cell lines at different proliferation stages using the Cyranose320 e-nose device. Also presented are results of five different classifiers for the e-nose to perform the VOCs classification. To the best of author knowledge, this paper also presents a novel work by investigating the use of Naïve Bayes (NB) and One versus All-Support Vector Machine (OVA-SVM) to classify the VOCs emitted by the in-vitro cell lines using e-nose. Table 2 shows the parameters used in this study.

**Table 2:** List of sensor array used, cancer cell lines, cell growth time considered for measurement (incubated period) and type of matrix used in this work

Sensor Type	Cell lines	Control	Incubations (hours)	Matrix Type	Statistical Approach
C320	NSCLC: A459 and Calu-3	WI38VA13, MCF7 and pure medium	24, 48, 72	Culture medium with cells	*Savitsky Golay filtering;* LDA,PCA, PNN, KNN, OVA-SVM, NB; 10-k-fold cross validation

The Cyranose320 is an array of 32 conducting polymer coated carbon black sensor-based e-nose and the pattern of change in the resistance of the sensor array is used to identify smells [37]. This feature can assist to detect even the slightest difference in headspace or complex volatile organic compounds (VOCs) emitted by the exhaled breath [38] or in vitro cultured cells [34, 39–41].The Cyranose320 was used to detect and discriminate the volatiles collected from the different cell lines with the aid of pattern recognition methods.

The VOCs collected were classified using different multiclass classifiers that best utilise the effectiveness of Cyranose 320 in distinguishing the lung cancer cells from control samples. GCMS-SPME analysis also performed for each sample. This pre-concentrated volatile compound extraction method was able to determine the specific compound emitted by each type of cells. The compounds were identified using NIST library and compared with e-nose data. Thus, the significance of this preliminary results and its support in the application in lung cancer clinical screening are discussed.

## Methods

### Cell culture preparation

Cancerous lung cell lines A549 (ATCC ® CCL-185™) and Calu-3(ATCC® HTB-55™), normal lung cell line WI38VA13 (ATCC® CCL75.1™) and breast cancer cell line MCF7 (ATCC® HTB-22™) were obtained from the American Type Culture Collection and being maintained at the Cell and Tissue Culture Engineering Lab (CTEL), Department of Biotechnology Engineering, IIUM. Table 3 shows the characteristics of the cell lines used in this project. Based on the Table 3, the A549 and Calu3 are representing same histology which is adenocarcinoma but claimed to be from different origin. Thus, the VOCs signature of both A549 and Calu3 will be also covered in this work.

**Table 3:** Characteristic of the cell lines

Cell Lines	Oncogene	Histology	Patient Type	Tissue Type	Growth Medium
A549	K-Ras mutation	Adeno-carcinoma	Male, 58 years Caucasian	Lung (epithelial cell)	DMEM
Calu-3	EGFR, K-Ras, TP53 and CDKNA genes mutation	Adeno-carcinoma	Male, 25 years, Caucasian	Lung (epithelial cell) from pleural effusion	EMEM
MCF-7	WNT7B mutation	Adeno-carcinoma	Female, 68 years	Mammary gland (epithelial cell) from pleural effusion	DMEM
WI38VA13	-	Normal cell	Female,Caucasian,3 months	Lung (Fibroblast)	DMEM

The A549, WI38VA13 and MCF7 cells were revived and cultivated in DMEM (Dulbecco’s Modified Eagles Medium) supplemented with 10% (*v*/v) FBS (Fetal Bovine Serum). Meanwhile, the Calu-3 cell line was grown in Eagle’s Minimum Essential Medium (EMEM) with 10% (v/v) FBS. The cells were grown in 25cm^2^ T-flasks and incubated in a carbon dioxide (CO_2_) incubator at 37°C/5% CO_2_ [22, 23, 36].

Upon reaching 70-90% confluence, the cells were harvested and then seeded into new flasks with an initial density of 1×10^5^ cells/ml in 5ml media for each cell line respectively. The culture condition was as reported in our previous work [39]. The blank mediums, DMEM (without cells) and EMEM (without cells) samples were also triplicates respectively as control samples and incubated together with A549, Calu-3, MCF7 and WI38VA13. Same cell culture preparation and environmental conditions were maintained for both e-nose and SPME-GCMS measurement. The odour samplings were taken after 24 h of incubation using SPME fiber (Divinylbenzene/Carbonexen/Polydimethylsiloxane), while for Cyranose320, the measurement commenced at 24th, 48th and 72nd hours of incubation.

### E-nose headspace sampling

**Fig. 1:**
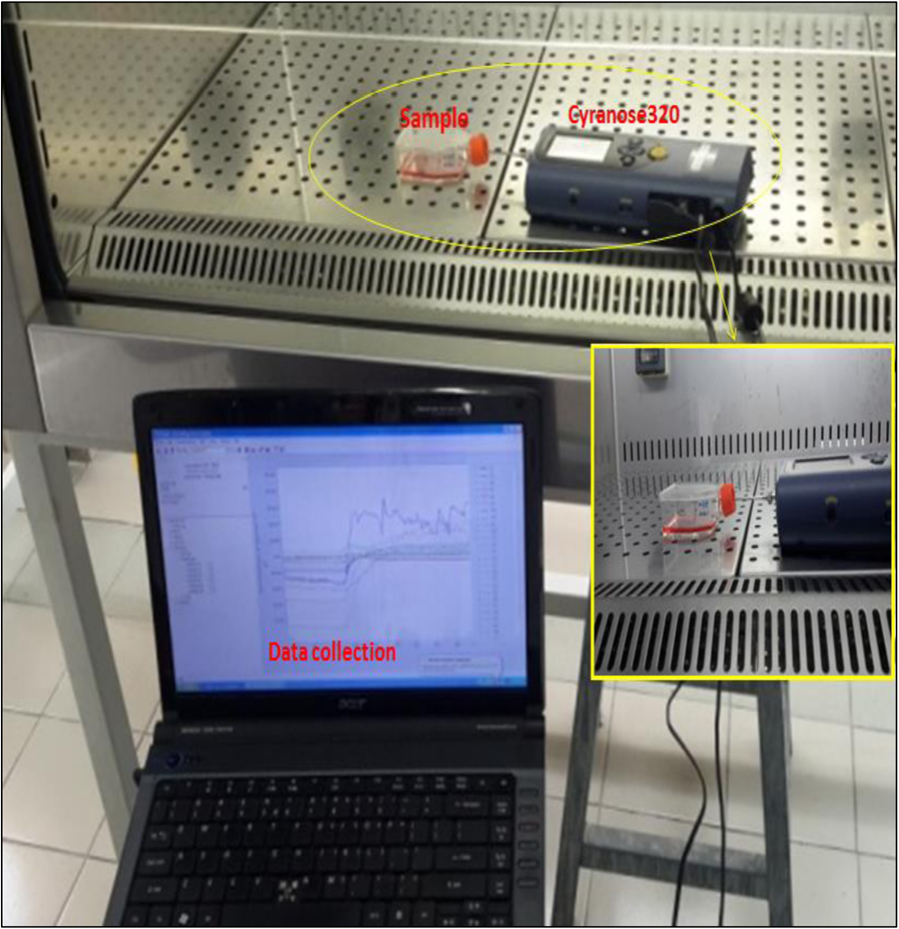
The Cyranose 320 Setup for Data Collection: Snout of Cyranose 320 was inserted into the T-25cm^2^ flask containing cell cultures

The prepared samples in fully sealed T-flasks were placed in the biosafety cabinet. Then the flasks were connected to the inlet of Cyranose 320 for data collection. The sampling setup using e-nose is shown in Fig. 1. Table 4 shows the configuration of the data collection process using Cyranose 320. The baseline purge was set to be at 10 s before data collection. The odour samples were drawn for 180 s to allow it to cover all the 32 sensors. This duration will enable all the sensors inside the Cyranose320 to detect the VOCs in the odour. The sniffing process was set to be repeated for 5 times.

**Table 4:** Parameter settings of the Cyranose 320 for data collection procedure [85]

Parameters	Time (s)	Pump Speed (cc/min)
Baseline Purge	10	160
Sample Draw	180	120
Snout Removal	3	-
1^st^Air Intake Purge	40	180
Filter: ON	-	-

The e-nose was set to be at 37°C (optimum temperature for culture growth)

### Data analysis

The collected data were then analysed using SPSS 17.0 and MATLAB R2012a to evaluate the e-nose performance. Each individual sample was described by a unique set of measurement known as features. The Cyranose 320 used in the work contains 32 conducting polymer sensors, and hence creates 32 features for each odour sample. Each feature forms a dimension in a space known as feature space. For each sample including the blank mediums, the experiments were replicated 3 times and each sniffing was repeated for 5 times at 24th, 48th and 72th hours respectively. For the e-nose analysis, each sample including blank mediums were replicated into three flasks, with datasets of two flasks used for training and the final one for testing. The sample datasets were divided into two parts and assigned as training and testing sets with a 2:1 ratio respectively. This study uses 18 different classes for classification purposes (total of six (6) classes multiplied by three varying incubation times).

**Fig. 2:**
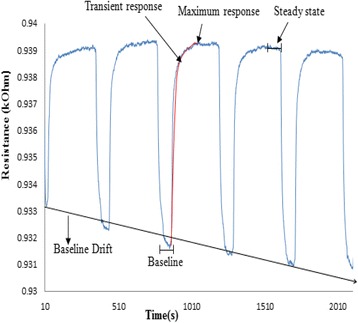
Example of five complete cycles of the feature space extracted from sensor 12 of the e -nose using A549 sample at 24th hour

**Fig. 3:**
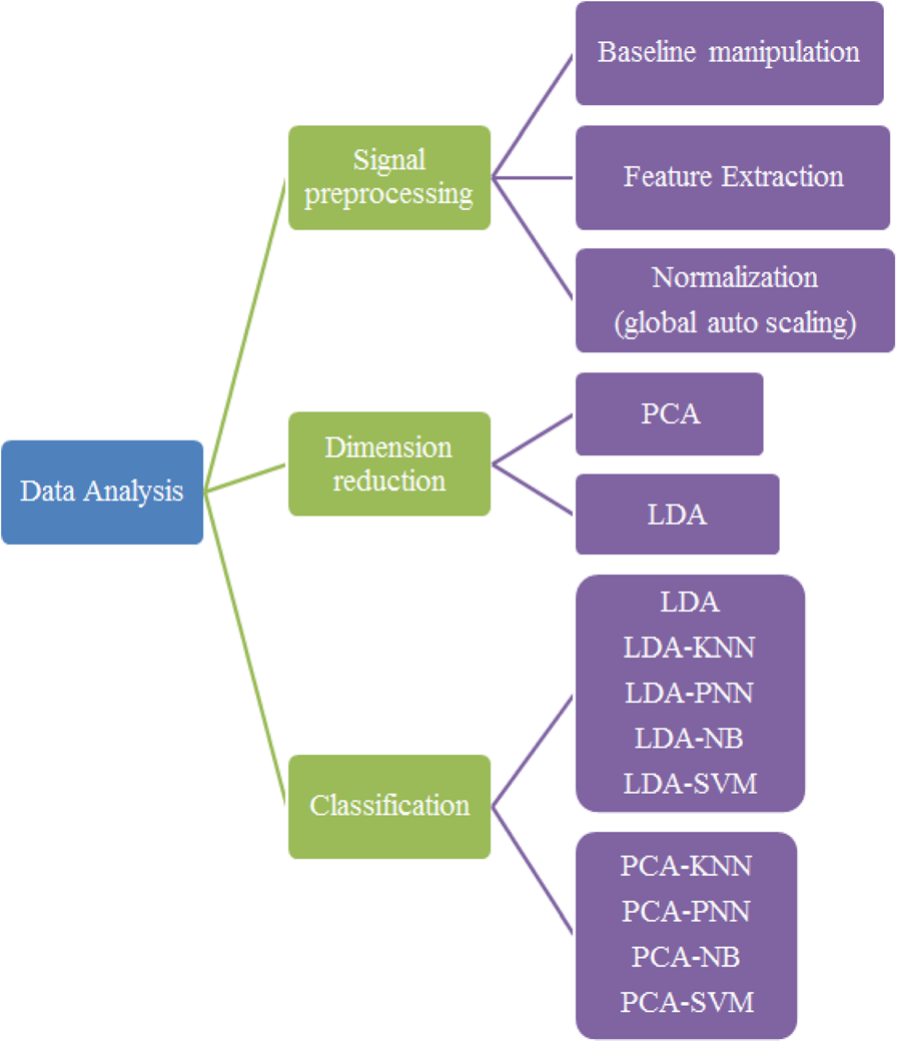
The block diagram of data analysis conducted using Cyranose 320

Figure 2 shows an example of five complete cycles of feature space from sensor 12 of the Cyranose 320. Figure 3 shows the block diagram of the summary of data analysis conducted in this study.

### Signal pre-processing

The Savitzky-Golay filter was selected to remove noise from the gas sensor signal while preserving the height, width, amplitude and overall profile of the response [37, 39]. The datasets were normalized using fractional difference method as in Eq. (1) [42]:

1 $$ \mathrm{dR}=\left(\mathrm{R}\hbox{--} \mathrm{Ro}\right)/\mathrm{R} $$

Where Ro is the baseline and the R is the steady state of the sensor response to the gas sample of the system. This fractional method helps to reduce the signal drift problem [43]. All data were further normalized using sensor auto scaling global method, scaled to zero mean and standard deviation of one [42, 44].

### Feature extraction

The consideration of features extraction is essential to point out the discriminating information that would aid the improvement of classification performance [38].

Principal component analysis (PCA) and linear discriminant analysis (LDA) are two commonly used feature extraction techniques [45, 46]. In this present study, both techniques were conducted to evaluate the best method for reducing dimensionality by preserving the minimum information about the dataset. Hence the component and discriminants from PCA and LDA respectively were used for class separability visualisation. The PCA provides unbiased projection, which gives better information on the clustering behaviour of each class, while LDA maximizes the intergroup variance and minimizes within group variance. Further, the LDA data was considered as the input for different classifiers. This LDA data able to provide the highest possible discrimination between different classes of data and help to classify the data accurately [47–50].

### Proposed classification algorithms

To date, various classification algorithms are proposed for cancer detection particularly those related to e-nose. In this study, the effectiveness and robustness of e-nose in distinguishing lung cancer cell lines were tested using several classification algorithms namely LDA with fisher criterion, K-Neighbour Neural Network (KNN), Probabilistic Neural Network (PNN), Naïve Bayes (NB) and Multi-class Support Vector Machine (SVM). The statistical significance of all 32 independent sensors was evaluated by comparing the mean score of 18 different groups using the Wilk’s Lamda method. A multi-class odour classification model (LDA-based classifier) was later proposed to evaluate the robustness of an e-nose system in classifying cancerous cell samples.

The LDA classification was conducted using leave-one-out approach for the error estimation. The fisher criteria was reported to be able to overcome the non-normally distributed data [51], hence being employed in this work.

PNN, which is defined as an implementation of Kernel discriminant analysis contains operations, which are organized into multi-layered feed forward network with four layers [52]. Although PNN algorithm required a large memory for training, it requires less training time [52, 53]. The spread value (σ) was determined using 10-fold cross validation and a value of 0.1 were obtained as appropriate for the dataset with acceptable classification accuracy [54].

On the other hand, KNN classification is known as the simplest classification which uses neighbour characteristics to determine the class of the data samples. This classifier is able to rapidly evaluate the unknown inputs by calculating the distance between a new sample and mean of training data samples in each class weight by their covariance matrices [23]. By considering the theoretical method the best k-value (one; 1) and the distance metric of Euclidean were selected as maximum accuracy obtained using these parameters [24].

Meanwhile, naïve Bayesian (NB) is a simple probabilistic classifier which applies Bayes’s theorem with naïve independence assumption. It is known as an efficient and effective classification technique to create models with predictive capabilities [55]as the algorithm does not have several free parameter settings, does not require large amounts of data for training and computationally fast in decision making [56, 57]. In this study, the NB classification with normal (Gaussian) was chosen and the prior probabilities for the classes specified to empirical.

Finally, SVM analysis is a linear classifier which is able to find the best separating line between two classes in higher dimensions [58]. However, the SVM can be directly used for binary classes only. For cases with more than two classes, the multi-class SVM can be implemented by dividing the single multiclass problem into multiple binary classification problems. There are three type of multi-class SVM, namely one versus all (OVA), one versus one (OVO) and Direct Acyclic Graph (DAG)-SVM [59]. The OVA based SVM was used in this work to classify the 18 classes. This classification was trained with RBF kernel functions which were obtained from optimization method [60]. Various pairs of box constraint (C) and sigma (σ) were tested for each dataset and the final obtained values were: C: 2^10^ and σ: 2^-3^for this dataset.

### Performance evaluation

The performance of each of the classifiers are presented using the accuracy (ACC) achieved. This is defined as the percentage (%) of correct classification over the total cases presented. However, since the accuracy alone might not give the best classification performance; sensitivity (SEN), specificity (SPE), precision (PREC) and Matthews Correlation Coefficient (MCC) measurements for each class were calculated to provide more relevant and interpretable information about the results [61, 62]. There are a few terms that are commonly used to measure the performance rate, namely, true positive (TP), true negative (TN), false positive (FP) and false positive (FP) [63].

The application of MCC in the multiclass case was originally reported in [64] which was used to measure the classification correlation. The value of MCC varies between -1 and 1 (where 1 is perfect prediction quality, while -1 is in the extreme misclassification of a confusion matrix and 0 specify random correlation) [62, 65]. This paper will report the accuracy, sensitivity, specificity, precision and MCC measures as well for all 18 classes for the best results.

### Gas chromatography mass spectrometry- solid phase micro extraction (GCMS-SPME)

#### GCMS-SPME headspace sampling

**Fig. 4:**
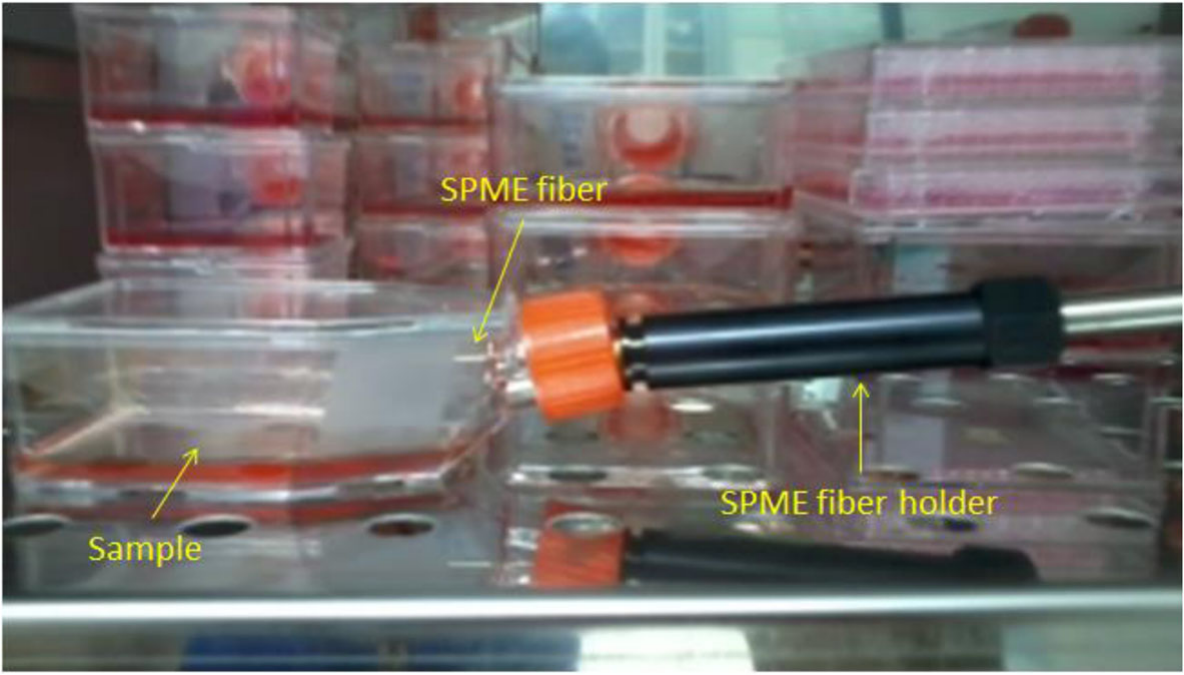
The GCMS-SPME odour sampling procedure. SPME coated needle was exposed to the headspace of cultured cell. The experiment was conducted in an incubator (37°C/5% CO_2_)

The SPME-GCMS was used to identify the headspace VOCs that were released by each type of cultured cell lines (A549, Calu-3, WI38VA13 and MCF7) and blank mediums. Preheated solid phase micro extraction (SPME) was used to collect the VOCs released from the cells. The inner needle, which is the fiber of SPME or known as Divinylbenzene/Carbonexen/Polydimethylsiloxane (DVB/CAR/PDMS), was used in this work. The DVB/CAR/PDMS coated fiber was chosen as it has been optimized to extract a wide range of molecular range of molecular weight of both volatile and semi volatile molecules [66]. The needle was exposed to headspaces of cell cultured in the 25cm^2^ T-flask for 15 min as shown in Fig. 4. At the end of the VOCs extraction time, the fiber was immediately inserted into GCMS Agilent 7890 sample point.

The DB-WAX capillary column (30 m x 250 μm x 0.25 μm) was used with the injector temperature of 250 °C to allow desorption of VOCs thermally. The oven temperature was initially set to be 50°C and held for 0.5 min, then ramped 10°C/minutes up to 180°C for 1 min and then again ramped 15°C per minute until it reached 250°C and held for 5 min. The carrier gas Helium flow rate was 1ml/min. The total analysis took 24.17 min to obtain the results. The MS analyses were done in full scan mode (TIC mode) with the scan range between 40 to 200 a.m.u and the electron impact ionization was done at 70eV to separate the compounds [30].

#### Identification of VOCs

The potential VOCs were only identified by using the spectral match in this study [29, 64]. The identity of each compound was determined using the Agilent Chem Station Software by searching on the “NIST” Mass Spectral Library 11 which provides the use of retention time and m/z of VOCs of interest. Each chromatograph was integrated and the peaks were matched and aligned in order to obtain a matrix that contains all peaks found in the whole set of measurements. The peaks or compounds that are missing in other replicate samples were eliminated. In this analysis, peaks less than 80% of the matching percentage to the NIST library (Qualitative) and peak area less than 3000 were excluded [27]. Those peaks identified as arising from column, empty flask and fiber (siloxanes) were excluded in this study [19, 29]. The significant differences on the relative abundances of identified VOCS were conducted using the t-test and considered significant at *P* < 0.05.

## Results

### E-nose performance

Table 5 shows a representative result of Wilk’s Lambda test of day 1 dataset to show the contribution of variation in the discriminant function (df). The functions with *p*-value less than 0.05 (*p* < 0.05) were chosen, as this corresponds to the ability of the function to discriminate the groups.

**Table 5:** The Significant test using Wilk’s Lambda for LDA

Test of Function(s)	Wilks' Lambda	Chi-square	df	Sig.
1 through 5	.000	8432.165	75	.000
2 through 5	.000	3116.215	56	.000
3 through 5	.033	1214.302	39	.000
4 through 5	.246	496.519	24	.000
5	.911	32.974	11	.001

*Df* different function, *Sig* significant value

**Fig. 5:**
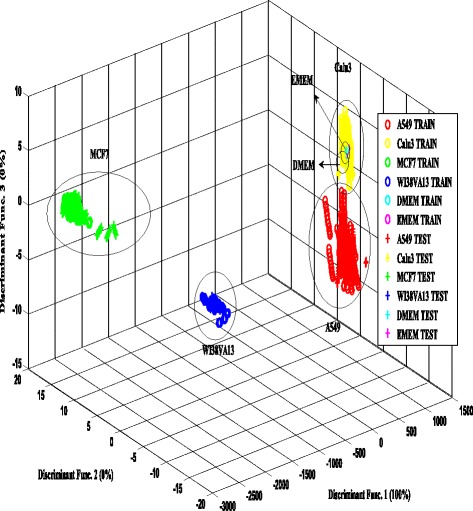
LDA plot of volatile compounds from cultured cells (combination of all 3 days). The separability of 4 types of cell lines and two different blank medium shows the effectiveness of the e-nose

**Fig. 6:**
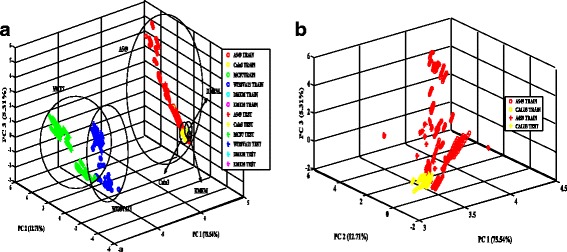
**a** PCA plot of volatile compounds of cultured cells (combination of all 3 days). The separability of 4 types of cell lines and two different blank medium shows the effectiveness of the e-nose. **b** PCA plot of volatile compounds of lung cancer cultured cells (combination of all 3 days). The separability of 2 types of lung cancer cell lines shows the effectiveness of the e-nose

Figures 5 and 6 show 3D scatter plots to visualize the variability between VOCs of cell lines detected by e-nose using LDA and PCA analysis respectively.

Based on Fig. 5, the result shows that the samples of A549, Calu-3, MCF7, WI38VA13 and blank mediums were well separated with 100% discriminant function. The test data samples were matched closely with the distribution of different groups of cell lines in the training data. A significant clustering between lung cancer cell, breast cancer and the control samples was observed. This indicates that the different cell lines are emitting different profile of VOCs and that the e-nose is able to detect these variations. Both of the non-small lung cancer cells, A549 and Calu-3 ,were observed to be very close together but with a distinct separation. The scores of other samples were well distributed within each group, respectively with visible separation for the combination of all days.

PCA was performed on the data and the eigenvectors and eigenvalues were calculated using correlation matrix. The eigenvectors of eigenvalue higher than 1.0 can be selected as principal components (PC) and value lower than 1.0 can be considered to be excluded, in this study, the first three PCs with eigenvalue higher than 1.0, were selected for dataset at 24th, 48th and 72nd hours. Based on Fig. 6a, the samples were observed to be well separated. The total percentage of principal components (PC1, PC2, and PC3) in the PCA analysis as shown in Fig. 6a is 93.56%, which indicates that the each of the cell lines are separable. In order to emphasise the ability of sensors to distinguish the different lung cancer type, the PCA plot for Calu-3 and A549 were enlarged in Fig. 6b. The sensors managed to distinguish the 2 types of lung cancer each other might be due to the specific VOCs emitted from the cell lines since the origin of the A549 and Calu-3 cells are from epithelium and pleural effusion, respectively.

However, based on the PCA grouping behavior, it is observed that the features within the group were separated spatially compared to the LDA. The clustering of A459 and Calu-3 (lung cancer cells) observed to be significantly separated from the MCF7 (breast cancer cell) and WI38VA13 (normal cell) clusters. Overall, the extracted feature by LDA indicates good separability of different samples. Thus the LDA-based features were used to test the four different classifiers.

### Classification results

The LDA-based features were used to test the four classifiers (LDA, PNN, KNN, NB and OVA-SVM) using 10-fold cross validation. The performance of these classifiers was measured by their accuracy, sensitivity, specificity, precision and MCC of training and testing data. The performances of the e-nose and the classifiers on differentiating the VOCs emitted by lung cancer from the control samples were evaluated by comparing of the performance each classifier.

Tables 6, 7, 8, 9, 10 show the of the classification results of five different classifiers performance in detecting each type of cell lines samples at three different times of incubation.

**Table 6:** Performance rate of Fisher-Linear Discriminant Analysis

Time (h)	Classes	Performance rate of LDA (%)									
		Train and Validate					Test				
		ACC	SEN	SPEC	PREC	MCC	ACC	SEN	SPEC	PREC	MCC
24	A549	90.86	98.41	98.93	98.41	0.91	96.89	98.61	98.88	98.44	0.94
	Calu3	94.84	94.38	95.50	98.52	0.75	95.15	95.26	96.18	97.52	0.74
	MCF7	94.32	94.92	95.50	88.89	0.80	95.01	94.72	96.30	90.32	0.82
	WI38VA13	94.73	93.20	95.72	92.86	0.89	95.56	94.50	94.53	92.99	0.89
	DMEM	93.25	93.33	95.18	87.69	0.75	92.96	92.63	95.75	93.17	0.78
	EMEM	90.22	92.25	91.33	84.39	0.70	90.96	89.84	95.65	83.01	0.67
48	A549	94.89	98.45	98.94	98.50	0.98	96.90	99.47	99.87	98.51	0.98
	Calu3	92.99	98.21	95.81	98.64	0.78	96.75	94.18	98.17	97.88	0.82
	MCF7	95.32	96.86	95.78	90.38	0.85	96.51	91.30	97.64	95.45	0.90
	WI38VA13	95.32	96.30	96.07	94.11	0.88	95.90	96.51	95.20	94.14	0.92
	DMEM	93.65	80.00	55.58	92.86	0.50	93.89	50.00	93.00	97.13	0.53
	EMEM	94.11	91.38	92.38	89.74	0.45	94.09	93.10	97.36	90.19	0.86
72	A549	94.91	98.62	98.97	98.50	0.98	96.92	99.54	99.89	98.63	0.96
	Calu3	94.04	98.67	94.52	98.34	0.86	97.41	95.33	98.04	97.71	0.85
	MCF7	95.95	96.98	96.00	92.32	0.92	97.12	92.91	97.76	95.71	0.95
	WI38VA13	95.59	97.05	96.57	94.23	0.93	96.34	96.54	97.30	94.70	0.88
	DMEM	93.52	83.08	96.00	94.30	0.65	93.68	52.31	93.21	97.25	0.71
	EMEM	94.44	88.89	94.80	73.85	0.52	91.85	88.89	95.21	90.75	0.37

**Table 7:** Performance rate of Naïve Bayes classifier

Time (h)	Classes	Performance rate of NB (%)									
		Train and Validate					Test				
		ACC	SEN	SPEC	PREC	MCC	ACC	SEN	SPEC	PREC	MCC
24	A549	96.87	97.00	99.32	99.45	0.97	99.39	99.27	99.68	99.47	0.95
	Calu3	97.56	96.13	98.41	99.35	0.95	99.34	96.25	99.54	99.49	0.94
	MCF7	98.70	97.44	97.68	95.79	0.93	99.23	98.31	99.29	90.63	0.84
	WI38VA13	98.83	94.91	95.67	99.22	0.96	99.34	94.23	99.65	94.23	0.88
	DMEM	92.86	61.52	98.09	70.59	0.38	97.10	73.68	98.63	77.78	0.53
	EMEM	91.71	60.22	96.77	56.12	0.33	92.81	73.02	94.18	46.46	0.87
48	A549	99.93	97.00	99.92	98.80	0.98	99.96	97.35	99.94	99.93	0.94
	Calu3	96.72	97.48	99.54	83.00	0.47	96.27	97.18	98.74	79.63	0.52
	MCF7	99.50	95.06	99.89	99.87	0.97	99.23	91.30	99.88	98.44	0.93
	WI38VA13	99.33	95.81	99.61	93.59	0.87	99.12	94.52	99.52	94.52	0.89
	DMEM	93.72	84.80	97.11	64.29	0.74	93.58	85.52	98.57	60.91	0.77
	EMEM	98.60	83.96	99.45	91.95	0.81	99.01	88.28	99.06	87.69	0.79
72	A549	99.93	97.00	99.92	98.99	0.98	100.00	100.00	100.00	100.00	1.00
	Calu3	96.41	95.90	96.24	64.75	0.36	96.78	90.00	97.25	69.23	0.43
	MCF7	99.75	97.00	100.00	100.00	1.00	100.00	100.00	100.00	100.00	1.00
	WI38VA13	99.70	97.00	99.68	96.19	0.94	99.78	97.96	99.88	97.96	0.96
	DMEM	92.56	87.53	96.85	91.75	0.66	93.19	80.00	97.01	91.23	0.67
	EMEM	97.25	90.75	97.51	73.77	0.59	98.47	94.44	98.73	82.26	0.69

**Table 8:** Performance rate of K-Nearest Neighbour classifier

Time (h)	Classes	Performance rate of KNN (%)									
		Train and Validate					Test				
		ACC	SEN	SPEC	PREC	MCC	ACC	SEN	SPEC	PREC	MCC
24	A549	99.08	97.67	99.17	99.50	0.91	99.47	97.97	99.20	99.60	0.91
	Calu3	99.87	99.98	99.86	97.75	0.96	92.70	99.84	99.07	99.57	0.98
	MCF7	99.01	99.77	99.54	99.65	0.97	99.04	99.87	99.92	99.80	0.97
	WI38VA13	99.41	99.48	99.37	99.33	0.95	98.80	99.40	98.80	92.30	0.89
	DMEM	99.54	92.47	97.32	96.35	0.92	93.80	87.50	97.38	96.45	0.94
	EMEM	99.93	98.85	96.37	91.02	0.88	87.10	71.40	98.40	85.20	0.92
48	A549	99.89	99.01	99.78	96.39	0.94	99.00	99.23	99.81	96.85	0.95
	Calu3	99.60	98.81	99.65	94.32	0.91	99.71	99.05	99.50	94.29	0.93
	MCF7	99.87	98.02	100.00	100.00	0.99	100.00	100.00	100.00	100.00	1.00
	WI38VA13	99.86	97.47	100.00	95.62	0.95	99.37	99.59	99.60	95.36	0.94
	DMEM	94.73	58.67	96.51	45.36	0.32	92.20	50.80	96.10	54.10	0.39
	EMEM	94.53	89.02	97.86	80.98	0.79	93.70	97.20	97.00	89.00	0.72
72	A549	99.94	98.98	99.93	98.98	0.93	99.90	99.89	99.83	99.93	0.96
	Calu3	99.60	96.41	98.98	98.79	0.96	99.75	99.58	99.55	95.20	0.95
	MCF7	100.00	100.00	100.00	100.00	1.00	100.00	100.00	100.00	100.00	1.00
	WI38VA13	100.00	100.00	100.00	100.00	1.00	99.80	99.67	100.00	100.00	0.97
	DMEM	96.46	78.04	95.72	86.34	0.63	86.80	100.00	85.80	86.36	0.66
	EMEM	99.60	100.00	99.58	94.12	0.91	94.20	18.50	100.00	100.00	0.40

**Table 9:** Performance rate of Probabilistic Neural Network classifier

Time (h)	Classes	Performance rate of PNN (%)									
		Train and Validate					Test				
		ACC	SEN	SPEC	PREC	MCC	ACC	SEN	SPEC	PREC	MCC
24	A549	99.62	98.77	99.22	99.59	0.92	99.65	99.08	99.39	99.69	0.94
	Calu3	99.89	99.98	99.89	98.05	0.97	99.87	99.89	98.70	98.91	0.96
	MCF7	99.11	99.80	99.58	99.69	0.98	99.04	99.87	99.92	99.80	0.97
	WI38VA13	99.50	99.57	99.43	99.44	0.94	99.61	99.62	99.47	99.49	0.96
	DMEM	99.57	95.97	99.31	97.33	0.93	99.80	95.99	99.48	97.75	0.95
	EMEM	99.90	98.93	96.47	91.02	0.89	99.93	97.14	95.14	91.22	0.89
48	A549	99.91	99.75	99.93	98.80	0.97	99.90	98.51	97.02	98.87	0.96
	Calu3	99.93	98.93	99.75	99.86	0.98	99.94	98.51	100.00	100.00	0.99
	MCF7	99.90	96.43	99.86	97.59	0.94	99.95	97.00	99.92	98.57	0.95
	WI38VA13	99.90	98.00	100.00	100.00	1.00	100.00	100.00	100.00	100.00	1.00
	DMEM	95.24	94.43	94.93	96.67	0.92	91.57	81.03	95.05	62.47	0.79
	EMEM	96.93	98.22	98.66	90.87	0.82	96.97	98.36	98.78	92.39	0.84
72	A549	99.93	100.00	99.93	98.99	0.97	100.00	100.00	100.00	100.00	1.00
	Calu3	99.94	98.90	100.00	100.00	0.99	99.69	100.00	99.67	95.24	0.93
	MCF7	99.92	97.00	99.91	97.73	0.93	99.97	97.54	99.68	94.23	0.92
	WI38VA13	100.00	100.00	100.00	100.00	1.00	100.00	100.00	100.00	100.00	1.00
	DMEM	91.24	94.41	95.80	88.11	0.70	91.66	94.62	96.01	88.57	0.68
	EMEM	97.73	58.53	98.90	94.82	0.67	97.99	58.99	98.97	95.63	0.69

**Table 10:** Performance rate of Support Vector Machine classifier

Time (h)	Classes	Performance rate of SVM (%)									
		Train and Validate					Test				
		ACC	SEN	SPEC	PREC	MCC	ACC	SEN	SPEC	PREC	MCC
24	A549	99.69	99.24	99.93	99.67	0.98	99.86	99.65	99.96	99.82	0.98
	Calu3	99.93	99.81	99.92	99.89	0.98	99.84	99.87	99.72	99.76	0.98
	MCF7	99.98	99.87	99.97	99.92	0.96	99.94	99.95	99.98	99.89	0.98
	WI38VA13	99.72	99.80	99.72	99.84	0.97	99.71	99.82	99.83	99.87	0.97
	DMEM	99.46	96.77	99.65	98.32	0.94	99.85	96.99	99.69	98.75	0.96
	EMEM	99.86	100.00	99.85	97.75	0.97	96.38	96.19	97.83	91.64	0.87
48	A549	99.82	99.72	99.94	99.30	0.97	99.97	99.79	99.97	99.76	0.97
	Calu3	99.79	99.70	100.00	100.00	0.98	99.47	99.64	99.88	99.89	0.98
	MCF7	99.99	99.45	100.00	100.00	0.99	100.00	100.00	100.00	100.00	1.00
	WI38VA13	99.86	99.89	100.00	100.00	0.99	100.00	100.00	100.00	100.00	1.00
	DMEM	97.52	97.83	97.49	92.00	0.81	92.62	76.07	94.55	41.86	0.78
	EMEM	100.00	100.00	100.00	100.00	1.00	99.12	100.00	99.06	87.88	0.80
72	A549	99.98	99.71	99.93	99.82	0.90	100.00	100.00	100.00	100.00	1.00
	Calu3	100.00	100.00	100.00	100.00	1.00	99.96	99.93	99.88	99.83	0.98
	MCF7	100.00	100.00	100.00	100.00	1.00	100.00	100.00	100.00	100.00	1.00
	WI38VA13	99.64	80.20	100.00	100.00	0.89	100.00	100.00	100.00	100.00	1.00
	DMEM	97.52	88.82	99.86	96.15	0.71	95.36	82.31	100.00	100.00	0.85
	EMEM	97.84	100.00	91.31	82.67	0.81	95.76	81.85	98.43	86.67	0.84

Tables 6, 7, 8, 9, 10 shows that three out of five LDA-based classifiers (SVM, PNN, KNN and NB) were able to achieve accuracy, sensitivity, specificity and precision of 90% while MCC has the value of 1 (high prediction quality). However, the OVA-SVM classifier gives the best results as compared to the other classifiers for classifying lung cancer cell lines volatile data. This algorithm shows high accuracy, sensitivity, specificity, precision and MCC in the testing phase. On the contrary, the LDA classifier has the least performance achieved and many samples were wrongly classified.

Although LDA-based OVA-SVM showed the best performance, the percentage of accuracy, sensitivity, specificity, precision and MCC values using PNN algorithm shows consistently high for every class. The prediction quality value (MCC) of DMEM using LDA-based PNN algorithm shows only 0.3 lesser than the SVM. To support this fact, a study conducted by F.Moderasi (2014), suggested that the PNN algorithm can be used as an appropriate alternative for SVM as the training process of the PNN algorithm is easier than SVM algorithm [67].

The performance of NB was observed to be less than SVM, KNN and PNN classifier because it is a generative classifier, and generally this classifier is not as accurate as the discriminative classifiers [68]. However, the NB is still preferred to be used for the medical diagnosis application because of it is simple to build, easy to train and able to deal with the missing information [56, 57]. According to K. Huang (2005), the NB performance can be improved by training the NB classifier in a discriminative way [68] .Thus, this method can be considered in future work to obtain excellent results from NB classifier.

When the LDA-based OVA-SVM performance rate was investigated according to samples at different incubation time, it was found that the classification accuracy rate improved significantly, achieving approximately 99% for the growth features of 24th-hour incubation period. The performance rate was observed to also improve for samples at 48th and 72nd-hour of cell growth. These may indicate that the VOCs of each sample increased with prolonged incubation periods.

The low performance of OVA-SVM for the 24th-hour compared to the 2nd day data may due to the insufficient time for the metabolites or compounds to be released by the cells to into the headspace. This may also happen due to relatively low cell numbers which cause the lower production of VOCs compared to the 48th and 72nd-hour of incubations. This corresponds to a previous study on in-vitro lung cancer cells by Smith. D (2003), where a number of compounds in the headspace are directly proportional to number of cells. This problem can be overcome using more concentrated cell seeding that might also help the differentiation between the other cell lines at an early stage of growth [69].

### Identification of the VOCs of lung cancer cell lines and normal cell lines by SPME-GCMS analysis

The VOCs related to lung cancer cell metabolism were investigated using SPME-GCMS analysis. The headspaces of cultured lung cells have been compared to the headspace of medium with breast cancer cells, the normal lung cells and without cells, respectively. The complete list of identified VOCs, based on the average peak of total chromatograms of three replicates of each sample is tabulated in Table 11. These 32 selected compounds are supposed to emitted from the both background culture media and the metabolic activity of the cells.

**Table 11:** Compounds detected from the headspace of in vitro cultured cell lines (>80% of the NIST matching percentage)

Retention Time (min)	Library/ID	Summary of All Substances Identified by Spectral Match						CAS number
		A549	Calu-3	MCF7	WI38VA13	DMEM	EMEM	
3.33	Amphetamine	-	-	-	+	-	-	300-62-9
3.44	Decane	+	+	+	-	-	-	124-18-5
4.25	Ethylbenzene	+	+	+	+	+	-	100-41-4
4.95	O-Xylene	-	-	-	+	-	-	95-47-6
5.30	Propylbenzene	+	+	+	+	+	-	103-65-1
5.51	1-Ethyl-2-methylbenzene	+	+	+	+	-	-	611-14-3
5.74	Styrene	+	++	++	++	++	-	100-42-5
6.18	Dodecane	-	+	+	-	-	-	112-40-3
6.28	1,2,4-Trimethyl-benzene	-	-	-	+	-	-	95-63-6
6.57	Trimethyl[4(trimethylsilyl)butoxy]silane	+	+	+	+	+	-	7140-91-2
7.52	Cyclohexanol	+	+	+	+	+	-	108-93-097
7.64	Decanal	+	-	-	-	-	-	112-31-2
7.68	Nonanal	+	-	-	+	-	-	124-19-6
7.69	3,4-Dimethylheptane	-	-	+	-	-	-	922-28-1
8.02	2,4-DimethylUndecane	-	-	-	+	-	-	17312-80-0
8.33	1,3-Bis(1,1-dimethylethyl)benzene	+	+	+	+	+	-	1014-60-4
8.67	Tetradecane	+	-	+	+	+	-	629-59-4
8.74	2-Ethyldodecanol	-	+	-	-	-	-	19780-33-7
8.75	2-Ethylhexanol	-	+	-	+	-	-	104-76-7
9.00	Benzaldehyde	+	-	+	+	+	-	100-52-7
10.25	Dimethylsilanediol	+	+	+	+	+	+	1066-42-8
10.55	Acetophenone	+	-	+	+	+	-	98-86-2
10.60	Ethanedioic acid, bis(trimethylsilyl)ester	+	+	+	+	+	-	18294-04-7
10.88	2-Ethyl-1,3-dimethyl-benzene	-	+	+	-	-	-	2870-04-4
10.92	1-Methyl-2-Pyrrolidinone	+	-	+	-	+	-	872-50-4
11.30	Heptadecane	-	-	-	+	-	-	629-78-7
11.22	Heneicosane	+	+	-	-	-	-	629-94-7
11.30	Hexadecane	-	-	+	-	-	-	544-76-3
11.37	2-(Aminooxy)-Propanoic acid	-	-	-	-	-	+	2786-22-3
11.48	3-Methyl-3-Hexanol	-	-	-	-	-	+	597-96-6
11.55	Methoxyphenyl_Oxime	+	+	+	+	+	+	1000222-86-6
12.96	2-Phenyl-2-Butanone	-	-	+	-	-	-	2550-26-7-97

++: Percentage of peak area more than 50%; +: percentage of peak area less than 50%; - : not detected (peak area < 1%)

Statistical significance of the relative abundances of the VOCs released from the lung cancer cell lines and the blank mediums have been evaluated using the t-test by considering *p* value less than 0.05 as statistically significant. This analysis conducted to eliminate confounding VOCs which are due to the different substrates rather than to the cell metabolism. The results were shown in Table 12. The same analysis also has been conducted on the VOCs released by the different cancer cell (MCF7) and the normal lung cancer (WI38VA13). The compounds and their significant differences have been tabulated in Table 13.

**Table 12:** VOCs discriminating the headspace of lung cancer cell lines and blank mediums. Analysis of abundances of VOCS in the headspace of lung cancer cell lines using GCMS-SPME. VOCs increased (emitted) and or decreases (consumed) by lung cancer are reported with respect to blank medium. A *p*-value < 0.05 has been considered statistically significant

Trend	Library/ID	*P*-value
Increase	Decane	1.50E-5
	Ethylbenzene	0.052
	Propylbenzene	0.011
	1-Ethyl-2-methylbenzene	0.037
	Styrene	0.135
	Dodecane	0.033
	Cyclohexanol	0.102
	Decanal	0.001
	Nonanal	0.002
	1,3-Bis(1,1-dimethylethyl)benzene	0.084
	Tetradecane	0.506
	2-Ethyldodecanol	3.88E-5
	2-Ethylhexanol	3.52E-5
	Benzaldehyde	0.590
	Acetophenone	0.750
	2-Ethyl-1,3-dimethyl-benzene	0.036
	1-Methyl-2-Pyrrolidinone	0.319
	Heneicosane	3.35E-6
Decrease	Trimethyl[4(trimethylsilyl)butoxy]silane	0.101
	Ethanedioic acid, bis(trimethylsilyl)ester	0.107

**Table 13:** VOCs discriminating the headspace of lung cancer cell lines and control cell lines. Analysis of abundances of VOCS in the headspace of lung cancer, breast cancer and normal lung cell lines using GCMS-SPME. A *p*-value < 0.05 has been considered statistically significant

Compounds	*P*-values				
	A549/Calu3	A549/MCF7	A549/WI38VA13	Calu3/MCF7	Calu3/WI38VA13
Decane	0.326 (↑)	0.154 (↑)	1.04E-4 (↑)	0.127 (↓)	0.016 (↑)
Propylbenzene	0.507 (↑)	0.533 (↑)	0.104 (↓)	0.988 (↓)	0.071 (↓)
Dodecane	1.67E-6 (↓)	2.23E-5 (↓)	-	0.011 (↑)	1.67E-6 (↑)
Decanal	0.001 (↑)	0.001 (↑)	0.001 (↑)	-	-
Nonanal	0.002 (↑)	0.002 (↑)	1.17E-4 (↓)	-	2.21E-4 (↓)
3, 4-Dimethylheptane	-	2.75E-4 (↓)	-	2.75E-4 (↓)	-
2, 4-DimethylUndecane	-	-	0.017 (↓)	0.017 (↓)	-
2-Ethyldodecanol	3.88E-5 (↓)	-	-	3.88E-5 (↑)	3.88E-5 (↑)
2-Ethylhexanol	3.53E-5 (↓)	-	2.47E-7 (↓)	3.53E-5 (↑)	9.40E-5 (↑)
Heneicosane	0.334 (↓)	0.002 (↑)	0.002 (↑)	3.40E-4 (↑)	3.40E-4 (↑)

The (↑) and (↓) shows the trend of abundances increases and decreases in lung cancer cell line samples respectively

Among the 32 VOC compounds detected, 20 are related to the lung cancer cell lines. Out of these, 18 are observed to be significantly more in the headspace of lung cancer samples compared to the blank medium (Table 12). Out of those 18, nine were observed to be absent from the blank samples. This indicates that these nine VOC compounds have specific association with the lung cancer cell metabolism.

In order to eliminate the influence of VOCs of culture media on the VOCs of lung cancer, the VOCs that found exclusively in the blank medium (statistically not significant) have been removed in the further analysis aimed at studying the properties of cancer cell lines. Furthermore, the aromatic compounds such as styrene, dimethyl silanediol, benzene and ethylbenzene are more linked to the contaminants [19, 50, 70, 71], thus these compounds are also eliminated for further analysis.

Overall, the 11 VOCs identified as statistically significant in previous analysis for the discrimination between normal lung cell and breast cancer cell line. The abundances of each VOC related to lung cancer cells was compared to both lung cells and breast cancer samples and tabulated in Table 13.

As seen in Table 13, four VOCs, namely dodecane, decanal 2-ethyldodecanol and heneicosane, are specific to lung cancer cells. They are absent from the control samples. The VOC whose abundance significantly decreases in the lung cancer cells are propylbenzene, nonanal, 3, 4-dimethylheptane, 2, 4-dimethylundecane and 2-ethylhexanol. The decane was observed to be increases significantly in the cancer related cell samples compared to normal lung cell line, indicating this compound more related to cancerous volatile. These results indicated that the headspaces of lung cancer cell lines are characterized by a specific VOCs signature.

## Discussion

The VOCs analysis in the medical field offered a great alternative approach to cancer diagnosis. However, till date the use of VOCs analysis in the clinical approach is still limited due to the lack of validation of cancer related metabolites and sensing performance of VOCs sensors. In this work, the VOCs emitted by the 2 different lung cancer cell lines and the controlled cell lines, both breast cancer cell and normal lung cell lines were analyzed using the commercialized CP gas sensors (Cyranose 320) and GSMS-SPME. This work is highlighting the potential of these analysis techniques in providing meaningful information in the clinical application of lung cancer diagnosis. The Cyranose 320 e-nose used to analyze the headspace of conditioned culture cell lines (in-vitro) in the proliferative conditions for 3 days to discriminate the VOCs patterns released in the headspace of the cell lines during normal and proliferation stage. Results from the e-nose analysis highlighted that the cancer cell lines are able to classified with high accuracy using the VOCs patterns even at the early stage of cell proliferation (24th hours of incubation time).

The ability for the Cyranose320 to be able to discriminate the VOCs of the cell samples with high accuracy even at the 24th hour of incubation provides a motivation to perform GCMS-SPME analysis. This allows the identification of the specific VOCs that are associated with the cancer cell growth. This was achieved by comparing the VOCs from lung and breast cancer cells to those of the blank mediums. Comparison of the chromatograms indicated that there were significant differences between the cell culture samples based on several compounds. There are total four specific VOCs identified as lung cancer related volatile, namely, heneicosane, dodecane, 2-ethyldodecanol and decanal.

The GCMS result also shows that higher alkanes group; heneicosane was found in both lung cancer cell lines, A549 and Calu-3, statistically significant from the controlled samples. This indicates that the heneicosane has high potential to be the lung cancer related biomarker. There are studies claimed the heneicosane as a candidate of the biomarker from lung cancer patients breath [28, 72, 73]. However, the origin of heneicosane in lung cancer cell remains unclear.

Another compound with a higher alkane group known as dodecane was observed to increases significantly in Calu-3 during the incubation period. There are few studies on lung cancer biomarker suggested n-dodecane to be associated with lung cancer in adenocarcinoma tissues [29], patient’s breath, especially in EGRF mutated adenocarcinoma patient’s breath [74]. Dodecane also found to be related to breast cancer [75].

Among the detected VOCs, one specific compound, namely decane, which is also from the high alkanes group, was observed to be emitted by all of the three cancer cells. Similar results were obtained by Yishan. W and B G.Hyun. the decane is found in the lung cancer tissue of patients [29, 72]. Another study by Chen. X, using different lung cancer cells also found that decane to be one of the 11 compounds with higher concentrations compared to those of normal cells [76]. Decane also considered as a lung cancer biomarker in a patient’s breath [77, 78]. A significant difference found in the concentrations of decane in the patient’s breath before and after surgery [79]. Still, the origin of decane in breast cancer cell has never been reported in any previous studies.

According to a study by Meggie. H (2010), representative of hydrocarbon is reported as potential biomarker of lung cancer and suggested that these compounds are probably the outcome of oxidative stress [80]. The alkanes are mostly produced from lipid peroxidation by reactive oxygen species (ROS) supported by few studies stating that alkanes and methylated alkanes are found in lung cancer [50, 70, 71, 80] and breast cancer [31, 34, 81].

A specific VOC released by A549 cell lines distinguished this cell line from other cell lines and blank medium which is decanal. A study in 2011 reported that decanal was used as a biomarker to detect non-small lung cancer using electronic nose with 95% sensitivity and 70% specificity [82]. Decanal was used as one of the primary contributors to separate non-small cell lung cancer and small cell lung cancer as well, with 100% sensitivity and 75% specificity by Barash. O in a study conducted in 2012 [33]. Whereas, there is only one specific VOC, 2-ethyldodecanol has been emitted by Calu-3.

The obvious VOCs emitted by MCF7 cell in this study were 3, 4-dimethylheptane, hexadecane and 2-phenyl-2-butanone. This finding is in line with one study which found hexadecane in the breath of a breast cancer patient [31]. However, no previous published studies on volatiles from breast cancer have reported the existence of 3, 4-dimethylheptane and 2-phenyl-2-butanone. The normal cell WI38VA13 emitted four different VOCs which were Amphetamine, Xylene, 2, 4-dimethylundecane and heptadecane. The 2-ethydodecanal, 3, 4-dimethylheptane, 2-phenyl-2-butanone, Amphetamine, Xylene, 2-4-dimethylundecane and heptadecane have not reported to date as biomarker in any in-vitro studies. Thus, the significance of these compounds remains unclear. Besides, the measurement time for VOCs collection used was in contrast with previous studies, where the VOCs collected after 24 h of cell growth. This is to ensure the compounds were collected at proliferation stage.

Nonanal and 2-ethylhexanol from WI38VA13 cells were found to be significantly more than that from A549 and Calu-3. In contrast to results observed in this study, it has been reported that the detection of nonanal is significant [83, 84] and used to separate adenocarcinoma and squamous cell carcinoma [74]. As for 2-ethylhexanol, the results here corresponds to other previous studies on lung cancer detection, and was never found to be one of the biomarkers. This indicates that these compounds might have a specific association related to cell metabolism. The WI38VA13 cells also share aromatic compounds with DMEM, which might be the reason for the overlapping of DMEM group in the WI38VA13 in the PCA and LDA analysis as shown in Figs. 5 and 6a.

In summary, the VOCs that exist in lung cancer cell lines but not in the control samples and those which exists in higher concentrations in the former may be considered as possible biomarkers as shown in Table 14. Decanal, dodecane, 2-ethyldodecanal and heneicosane may potentially be used to discriminate lung cancer cells from other type of cancer or normal cell lines. Decane on the other hand can potentially be used as a specific biomarker for cancer. These findings suggested that the identified VOCs are able to offer more information regarding in-vitro cultured cell line metabolism and aid the determination of lung cancer using the electronic nose technology. In order to reduce the possibility of false positive results, it is crucial to creating libraries of biomarkers for each of cancer cells and normal cells. This can be achieved by performing various chemometric or multivariate analysis to validate the biomarkers of the cancerous and normal cells of interest.

**Table 14:** The potential lung cancer biomarkers detected in this study were compared to the in-vitro and in vivo results of previous studies which were found in Scopus database

Class Specific compounds (In this study)		Origin	Comparison with Literature			
			Cell lines	^a^Tissues	Breath	References
Alkanes Straight chain	Dodecane Heneicosane	Endogenous- lipid peroxidation by reactive oxygen species (ROS) s	✓	✓	✓✓	[28, 29, 72, 76, 78–80]
			-	✓	✓	[29, 74, 80]
			-	-	✓	[28, 72, 73]
Aldehydes	Decanal	Endogenous- peroxidation of omega3 and omega6 fatty acids (PUFAs), components of cell membrane phospholipids	✓	✓	-	[33, 80, 82, 86]
	Nonanal					
Alcohol	2-Ethyldodecanol	Endogenous- Hydroxylation of the lipid peroxidation biomarkers via cytochrome p450 enzymes	-	-	-	[80]

^a^Patient’s tissue

✓Compounds that were detected as lung cancer biomarkers in previous studies

- Compounds that have not been detected in the previous study

## Conclusion

This study presents the possibility of using VOCs as biomarkers for cancer cells. Specific VOCs are verified to be specific to cancer cells compared to of the normal samples. The headspace of in-vitro cultured cell lines were analyzed using a Cyranose320 e-nose consisting of an array of sensors and GCMS coupled with SPME. Several classifiers were used to validate the ability of the e-nose to discriminate the cancer cells to that of the normal samples and blank mediums, namely the LDA, NB, KNN, PNN and OVA-SVM. The investigation was carried out to identify cell lines VOCs at three different proliferation stages under a normal laboratory condition.

The results from this study shows that the Cyranose320 was able to discriminate the VOCs released by the various cancer and healthy cells as well as the blank mediums. The classifiers tested were able to perform high levels of accuracy. The LDA based OVA-SVM records the best performance with 100% successful classification, even at the early stage of cell growth (24th hours of incubation) and managed to maintain this performance at 48th and 72nd hours.

The VOCs pattern collected from e-nose results were validated by the GCMS-SPME. The results show that particular cell lines produced specific VOCs. This study provides a list of possible VOCs, which is believed, can be specific biomarkers for lung cancer, even at the 24th hour of cell growth. The potential list of VOCs obtained from this study was compared with the previous studies as shown in Table 14. This also concludes that the e-nose in conjunction with GCMS-SPME is able to be a non-invasive screening tool at an early stage. This is particularly useful for the clinician to understand in the event any occurrences of overlapping groups in the e-nose results.

Besides, this study also shows that the use of existing tools such as GCMS-SPME and e-nose-based gas sensor array system promises the potentials to improve the cancerous VOCs detection system by optimizing the sensor selections. The sensors with higher selectivity and sensitivity are essential in order to capture the specific biomarkers. Therefore, further studies on optimizing the sensor system and using in-vivo studies (e.g. using breath samples) are underway with the ultimate goal to develop a complementary tool for clinical testing.

## Abbreviation

A549: Lung cancer cell line
ACC: Accuracy
ANN: Artificial neural networks
Calu3: Lung cancer cell line
DMEM: Dulbecco’s modification of Eagle medium
EMEM: Eagle’s minimum essential medium
E-Nose: Electronic nose
FN: False negative
FP: False positive
GCMS: Gas chromatography mass spectrometry
GNP: Gold nanoparticles
HASM: Human airway smooth muscle
IBE: Immortal bronchial epithelium
KNN: K-Neural network
LDA: Linear discriminant analysis
LDCT: Low dose computed tomography
MCC: Matthews correlation coefficient
MCF7: Breast cancer cell line
MD: Mahalanobis distances
NB: Naïve bayes
NHDF: Normal human diploid fibroblast
NSCLC: Non-small cell lung cancer
OVA: One versus all
OVO: One versus one
PC: Principal component
PCA: Principal component analysis
PDMS: Polydimethylsiloxane
PNN: Probabilistic neural network
PREC: Precision
Rmax: Maximum value of response
Rms: Root mean square roughness
Rs: Steady state of response
SCLC: Small cell lung cancer
SEN: Sensitivity
SGF: Savitzky-Golay smoothing filter
SPE: Specificity
SPME: Solid phase micro extraction
SVM: Support vector machine
TIC: Total ion chromatogram
TN: True negative
TP: True positive
VOCs: Volatile organic compounds
WI38VA13: Normal lung cell line
